# Acknowledgment to the Reviewers of *Marine Drugs* in 2022

**DOI:** 10.3390/md21020071

**Published:** 2023-01-21

**Authors:** Marine Drugs Editorial Office

**Affiliations:** MDPI AG, St. Alban-Anlage 66, 4052 Basel, Switzerland

High-quality academic publishing is built on rigorous peer review. *Marine Drugs* was able to uphold its high standards for published papers due to the outstanding efforts of our reviewers. Thanks to the efforts of our reviewers in 2022, the median time to first decision was 13 days and the median time to publication was 32 days. Regardless of whether the articles they examined were ultimately published, the editors would like to express their appreciation and thank the following reviewers for the time and dedication that they have contributed to *Marine Drugs*:
Abate, MarioLi, JiyunAbbastabar, HosseinLi, KechengAbdalla, ShtaywyLi, Min-HuiAbdelfattah, MohamedLi, PengchengAbdelkader, HamdyLi, RongfengAbdelkader, Noha F.Li, XinAbdel-Mogib, MamdouhLi, XuwenAbdelmohsen, Usama RamadanLiao, Hsiang-RueiAbe, IkuroLiaw, Chih-ChuangAberturas, JavierLicea, AlexeiAboal, MarinaLigabue-Braun, RodrigoAboul-Soud, Mourad A. M.Lijuan, LongAdachi, KohsukeLima Ribeiro, MarceloAddison, RussellLin, HaishuAgòcs, AttilaLin, LigenAguilar, Manuel B.Lin, Tung-YiAh Young, ParkLin, Yu-HsinAhmad, IffatLin, Zhen-JianAhmed, Mohammed MuqtaderLioe, Hanifah NuryaniAirs, Ruth LouiseLitaker, WayneAkkarasamiyo, SunisaLittle, PeterAkopova, TatianaLiu, LanAlarif, Walied MohamedLiu, LingAlbericio, FernandoLiu, Shing-HwaAlbini, AdrianaLIU, ShuchengAlduina, RosaLiu, YonghongAllen, ScottLiu, Yun-BaoAl-Massarani, ShazaLiu, ZhiqingAl-mourabit, AliLiuzzi, Grazia MariaAl-Sabi, AhmedLlaver, MauricioAlvarez, Alejandro J.Llewellyn, Lyndon E.Alvarez-González, Carlos AlfonsoLongato, Giovanna BarbariniAmaral, Adérito J. R.López, Lucía MéndezAmato, AlbertoLopez, MichelAmato, JussaraLópez-López, ManuelAmen, YhiyaLordan, RonanAmer, ShimaaLoura, Luís M. S.Amorim, ManuelaLouro, RicardoAmoutzias, GrigorisLu, JiAndrée, BirgitLu, Mei-ChinAndrianasolo, EricLu, ZhaoyongAngelini, PaolaLucchetti, JacopoAntoniassi, RosemarLuceri, CristinaApicella, AntonioLučić, BonoAraújo, Rafael G.Lukic, IvaArmeni, TatianaLuo, SulanArteaga, Jesús FernándezLuo, XiaoweiArthur, GilbertMa, JunyingArtyukhin, AlexMacÍas Sánchez, Antonio JoséArumugam, MuthuMacias, Maria J.Arumugam, Vijaya AnandMacMillan, John B.Asai, DaisukeMadgett, TraceyAsanka Sanjeewa, KalukapugeMadhukar, BurraAsano, KazuhitoMadoui, Mohammed-AminAshour Fikry, MohamedMaeda, HayatoAtanase, LeonardMaggi, Leonard B.Atanassova, MiroslavaMahomoodal, FawziAvetissov, IgorMakris, Antonios M.Avilés, Francesc XavierMalik, AdeelAzam, FaizulMalyarenko, Timofey V.Azimi, BaharehMambu, LengoAzmi, MohamadMancheño, JoséBaby, André RolimMandal, Tapas K.Badiale Furlong, ElianaManera, MaurizioBaghban, NedaMangoni, AlfonsoBagheri, AliMannino, GiuseppeBailly, ChristianMansour, AbdallahBaindara, PiyushManzo, EmilianoBalcerczyk, AnetaMaoka, TakashiBalestri, FrancescoMarasco, DanielaBallarin, LorianoMarini, Herbert RyanBanday, Abid H.Mariychuk, RuslanBanskota, Arjun H.Markou, GiorgosBao, RuiyangMarra, AlbertoBarajas-Solano, AndresMartínez-Alvarez, OscarBaranenko, DenisMartín-Illana, AraceliBarba, Francisco J.Martins, SoniaBardakova, Kseniia N.Maruyama, HirokoBarone, RobertoMassiot, GeorgesBarra, LuciaMastino, AntonioBarreto, MariaMasuzaki, RyotaBartosz, GrzegorzMatafome, PauloBarzkar, NooraMatsubara, TakumaBasco, LeonardoMatsugo, SeiichiBastos, VerónicaMatsuura, HiroshiBatista, IrineuMattei, CésarBattino, MaurizioMatthews, Jennifer L.Batubara, IrmanidaMáximo, PatríciaBechthold, AndreasMazur, MarcelinaBedini, EmilianoMcClintock, JamesBeedessee, GirishMcFadden, SandraBeemelmanns, ChristineMcgrath, SaraBener, MustafaMeana, ClaraBenhabbour, Soumya RahimaMedeiros Borsagli, Fernanda Guerra LimaBenítez-Mateos, Ana I.Mehariya, SanjeetBenkendorff, KirstenMehra, Neelesh KumarBenko, AleksandraMei, Kuo-ChingBéraud-Dufour, SophieMelcer, Michael E.Berdyshev, Evgeny V.Meléndez-Hevia, EnriqueBergandi, LoredanaMelis, AnastasiosBerger, StefanMeliţ, Lorena ElenaBernardo, AntoniettaMenaa, FaridBertrand, SamuelMenchinskaya, Ekaterina S.Besednova, Nataliya N.Mendiola, JoseBetancur Ancona, DavidMeng, Ling-HongBevins, CharlesMenzel, LorenzoBezerra, Raquel PedrosaMertins, OmarBhuyar, PrakashMeslet-Cladiere, LaurenceBian, XiaoyingMessina, ConcettaBianco, ArmandodorianoMete, YilmazBickmeyer, UlfMi, Fwu-LongBideshi, DennisMia, SobujBinda, ElisaMicael, JoanaBiondi, NatasciaMichalak, IzabelaBisio, AngelaMiguel, Sónia P.Bišová, KateřinaMikkelsen, MariaBitchagno, Gabin Thierry MbahbouMinato, Ken-ichiroBlachowicz, AdrianaMinto, RobertBlanco, JuanMiricescu, DanielaBlomster, JaanikaMitchell, MiguelBobis, OtiliaMitić-Ćulafić, DraganaBoca, SandaMititelu, MagdalenaBocian, AleksandraMiyachi, HiroyukiBoggia, RaffaellaMiyamoto, KojiBonadies, IreneMladenovic, MilanBorbély, JánosMoeini, ArashBorges Dos Santos, Rui M.Mohamed, GamalBorges-Argaez, RocioMohamed, HassanBorymska, WeronikaMohanta, Yugal KishoreBoschi-Muller, SandrineMokrejš, PavelBosso, AndreaMolero, GloriaBossowska, AgnieszkaMolinaro, AntonioBotana, Ana MaríaMonge, ClaireBoudreau, Paul D.Moni, LisaBourguet-Kondracki, Marie LiseMoniuszko-Szajwaj, BarbaraBoyom, Fabrice FekamMonteiro, PedroBraganca, Judith M.Montero, OlimpioBravo, Susana B.Montesano, DomenicoBrecker, LotharMonti, Maria ChiaraBrown, Alan B.Moradi, SaraBrown, LindsayMorales, Ana Maria SimonetBrunetti, LuigiMorales-González, José AntonioBruno, InesMoreira, Daniel CarneiroBryan H., BellaireMoreira, SusanaBryndal, IwonaMoreno, Juan JoséBucciarelli, Gary M.Morita, NaokiBuchaim, Rogério LeoneMoroño, ÁngelesBūdienė, JurgaMoser, GleyciBuglak, AndreyMouga, TeresaBugni, TimMouncey, Nigel J.Bukvicki, DankaMoura, Nuno M. M.Bule, PedroMourão, Paulo A. S.Bustillos-Guzmán, JoséMoustafa, GaberButler, MichaelMozumder, Md. Salatul IslamByadgi, Omkar VijayMüller, Werner E. G.Byun, Woong SubMulloy, BarbaraCain, Ricky M.Munir, NeelmaCalarco, AnnaMurata, KazuyaCalcinai, BarbaraMurphy, Patrick J.Calcutt, MichaelMurray, Thomas F.Cammarata, MatteoMuschiol, JanCampos, Joaquín MaríaNafie, Mohamed S.Campos, Pierre-EricNagao, KojiCampos-Montiel, Rafael G.Naimi-Jamal, MRCañada, FranciscoNakagawa, HiroshiCannataro, RobertoNaletova, IrinaCapon, RobertNam, Sang-JipCaporale, AndreaNarayanankutty, ArunaksharanCarballeira, Nèstor M.Narciso, LauraCardá, MiguelNasr, FahdCardenas, ConstanzaNasr, MahmoudCarmeli, ShmuelNatalia, CastejónCarotenuto, RosaNathanailides, CosmasCarreon, LauraNehira, TatsuoCarrieri, AntonioNelson, DavidCarvalho, PauloNesic, AleksandraCascales, José Javier LópezNeumann, JakeCasertano, MarcelloNg, Wei LongCasillo, AngelaNguyen, BinhCastellano, ImmacolataNgwa, Che JuliusCastro, José Javier FernándezNicolau, ElodieCastro, LeandroNicoletti, RosarioCatanzano, OvidioNicolò, Marco SebastianoCaturano, AlfredoNielsen, Vance GirardCeccarelli, GabrieleNiemand, JandeliCélio José, Castro-JuniorNieri, PaolaCervellati, FrancoNieto, Marcelo J.Ćetković, HelenaNirmal, NileshChakrabarti, SubhadeepNoman, EfaqChan, Fan LiangNorici, AlessandraChandra-Hioe, Maria VeronicaNorte, ManuelCharoensutthivarakul, SitthivutNorton, RayChekanov, KonstantinNoshita, ToshiroChen, BeiNovaković, Miroslav M.Chen, Hsin-YuanNovelli, AntonelloChen, SenhuaNowak, DorotaChen, XaolinNunes, NunoCheng, Juei-TangNuñez-Vazquez, ErickCheong, Kit LeongNycz, JacekCHEW, Kit WayneObluchinskaya, Ekaterina D.Chianese, GiuseppinaObruca, StanislavChiang, Chih-ShengOchiuz, LacramioaraChiang, Meng-TsanOfir, RivkaChiulan, IoanaOh, Dong-ChanChizhov, Alexander O.Ohshimo, KeijiroChoi, Hae WoongOhshiro, TakashiChoi, Jeong JuneO'Keefe, BarryChoudhary, DharmendraOliver, OzohanicsChronopoulou, EvangeliaOmar, Syed HarisChugunova, ElenaOniszczuk, AnnaChutrakul, ChanikulOpatz, TillCirillo, GiuseppeÖrdög, VinceColombo, DiegoOrisakwe, Orish EbereCopié, ValerieOrlikova, BarboraCorrea, S. G.Oshiro, NaomasaCorreia, Ilídio J.Osidak, EgorCorreia-da-Silva, MartaOstrovsky, Mikhail A.Corsaro, Maria MichelaOstrowska, KingaCosta, Pedro M.Otero, PazCostantino, ValeriaOu, Shi YiCostanza, BaldisserottoOuyang, JianmingCotas, JoãoOves-Costales, DanielCoulombe, BenoitOzeki, YasuhiroCovington, Brett C.Pacheco, RitaCowan, Ashton KeithPacker, MikeCrisan, LuminitaPadri, MohamadCristiano, LuigiPaduchová, ZuzanaCristiano, Maria ChiaraPaliwal, ChetanCroce, Anna CletaPampalakis, GeorgiosCrüsemann, MaxPan, HongmiaoCuellar, SaraPangestuti, RatihCueto, MercedesPannkuk, EvanCui, QiuPanzella, LuciaCustodio, LuisaPark, HyunCutignano, AdelePark, JunsooCuttitta, AngelaParrino, BarbaraCzernel, GrzegorzParsons, PeterD’Ambrosio, MarioPastene-Navarrete, Edgar R.D’Errico, StefanoPastor, NinaDaege, Haile FentahunPathom-aree, WasuDahiya, RajivPatruno, AntoniaDai, YuminPavadai, ParasuramanDalisay, Doralyn S.Pavao, Mauro SergioDamalanka, VishnuPavic, AleksandarDamiani, DanielaPearce, NorrieDanac, RamonaPelin, MarcoDandekar, AdityaPeng, Hsien-YuDanel, Krzysztof S.Peral, María J.Danish-Daniel, MuhdPereira, DavidDannenberger, DirkPereira, LeonelD'Argenio, ValeriaPereira, Sara B.Darie-Nita, Raluca NicoletaPérez Puyana, Víctor ManuelDarwiche, NadinePérez, José AntonioD'Auria, Maria ValeriaPerez, Juan J.D'auria, ValeriaPérez-Correa, José RicardoDave, DeepikaPerez-Stable, CarlosDaza-Navarro, PaulaPérez-Victoria, IgnacioDe Almeida, José RafaelPerveen, ShaguftaDe La Calle, FernandoPestov, AlexanderDe La Cruz, Armah A.Peter, MartinDe Lera, Angel RPetre, Brînduşa AlinaDe Los Reyes, CarolinaPetruczynik, AnnaDe Luca, DanielePiersanti, AriannaDe Souza, Alana GabrieliPiggott, Andrew M.De-Bashan, Luz E.Pilgaard, BoDefeudis, GiuseppePimentel, FilipaDegl'innocenti, DonatellaPinteus, SuseteDelbarre Ladrat, ChristinePinto, Diana CláudiaDeli, JózsefPiras, Anna MariaDella Sala, GerardoPisklak, Dariusz MaciejDellaGreca, MarinaPistarà, VenerandoDembitsky, ValeryPitsinos, EmmanuelDemirel, ZelihaPlascencia-Jatomea, MaribelDemori, IlariaPlastina, PierluigiDerkatch, SvetlanaPlayford, Martin P.Dermitzaki, EiriniPlisson, FabienDesmaële, DidierPlonka, PrzemyslawDey, KamolPoirier, DonaldDhasmana, AnupamPongrakhananon, VarisaDi Dato, ValeriaPopescu, IrinaDi Paola, LuisaPoulin, RemingtonDi Stefano, AntonioPozzolini, MarinaDi, XiaxiaPratsinis, HarrisDíaz, Luis EduardoPratt, StevenDiaz-Marrero, Ana R.Priel, AviDiederich, MarcPrieto, Miguel A.Ding, Zhong-TaoPrieto, Miguel ÁngelDinica, Rodica-MihaelaPrieto-Davó, AlejandraDinos, George P.Pristaš, PeterDiomede, FrancescaProsekov, AlexsandrDiVerdi, Joseph A.Prousis, KyriakosDjuris, JelenaPtaszyńska, NataliaDobrzynski, PiotrPucciarelli, SandraDolmatov, Igor Yu.Purohit, RiturajDomínguez, HerminiaPyka, AlinaDomínguez-Álvarez, EnriqueQian, Zhong-JiDomonkos, IldikóQu, XiaozhongDonadio, GiulianaRabbani, GulamDonato, Rosario FrancescoRacovita, RaduDong, PingRadwan, Mohamed O.Donnini, SandraRagab, AmanyDooley, HelenRahman, AzizurDračínský, MartinRahman, M. AzizurDrasar, Pavel B.Rai, AkhileshDu, ChenyuRaja, RathinamDu, LinRajski, Scott R.Duan, DelinRallis, MichailDufossé, LaurentRamesh, Chatragadda H.Dugal-Tessier, JulienRamos, Ricardo MartinsDuggan, Brendan M.Ramya, Thirumalai Nallan ChakravarthyDuong, Thuc-HuyRao, K.v.bhaskaraDuran, Verónica RojasRateb, MostafaDurán-Riveroll, LorenaRay, PriyankaDurek, ThomasRazafindralambo, Hary L.Dutertre, SebastienRebuffat, SylvieDutot, MélodyReczyńska, KatarzynaDyshlovoy, SergeyReeve, VivienneEbada, SherifReggelin, MichaelEckhard, UlrichRegla-Nava, Jose A.Eduardo Hernandez-Sandoval, FranciscoRein, KathleenEfremenkova, Olga V.Reis-Costa, PedroEhrlich, HermannRekha, P. D.Eilertsen, Karl-ErikRendueles, ManuelEinarsson, HjorleifurRescifina, AntonioEker Develi, ElifRevuelta, JuliaEl-Aasr, MonaRey, FelisaElango, JeevithanReyes, FernandoElbendary, AshrafRho, Jung-RaeElhady, SamehRibeiro, Teresa GonçalvesEl-Kersh, DinaRiccio, RaffaeleElsabee, Maher Z.Rivas, LuisEl-Sawy, EslamRivera-Monroy, Zuly JennyEl-Sayed, IbrahimRivera-Pérez, CrisalejandraEl-Sayed, Wael A.Robertson, Deborah L.El-Shazly, MohamedRocha, HugoEl-sheekh, MostafaRochefort, Gael Y.Elyashberg, MikhailRodolfo, CarloErdoğan, AyşegülRodrigo, AnaEsposito, GiuseppeRodrigues, Clara F.Es-Safi, Nour EddineRodrigues, FranciscaEssafi-Benkhadir, KhadijaRodrigues, Maria JoãoEstevez, PabloRodríguez Romero, AdelaEtxebeste, OierRodríguez, JaimeFacundo-Joaquin, Marquez-RochaRodríguez-Canul, RossanaFaenza, IreneRodríguez-García, IgnacioFalcó, AlbertoRodriguez-Monroy, MarcoFareed, JawedRodriguez-Troncoso, Alma PaolaFeás, XesúsRoje, MarinFedosov, AlexanderRok, JakubFelgueiras, HelenaRoman-Blas, Jorge A.Fendri, ImenRomano, GiovannaFenical, WilliamRosales Martínez, AntonioFernandes, ElizabethRoss, Ian L.Fernandes, PedroRossez, YannickFernández-Acero, Francisco JavierRougé, PierreFernandez-Lafuente, RobertoRoullier, CatherineFernando, Ilekuttige Priyan ShanuraRoy, AnuradhaFerreira, MarceloRoy, Biswajit GopalFields, Francis J.Rubio Alonso, JuanFigadere, BrunoRuiz-López, Mario AlbertFigueiredo, Marxa L.Ruzik, LenaFigueroa, FelixRyu, BomiFigueroa, MarioRywińska, AnitaFisher, Jed F.Saaby, LasseFitton, Janet HelenSaberi-Riseh, RoohallahFlematti, Gavin R.Saengkrit, NattikaFlórez-Fernández, NoeliaSaha, SatyajitFol, MarekSaha, SubhasishFonseca, Roberto J. C.Sahyon, HebaFormato, MarilenaSajkiewicz, PawełForzato, CristinaSakai, ShinjiFousteris, ManolisSakakibara, HiroyukiFraguas-Sánchez, Ana IsabelSalamone, MonicaFrancuz, JovanaSalerno, SimonaFrank, Craig L.Salim, AngelaFrau, JuanSalomon, ChristineFredotović, ŽeljanaSalom-Roig, Xavier JFreysdottir, JonaSalucci, SaraFujii, YukiSaluja, RohitFujimori, KoSalvador-Reyes, LilibethFujita, MasakiSalvatore, Maria MichelaFumagalli, LauraSamara, PinelopiFuwa, HaruhikoSamuel, AbaldeFuwa, HaruikoSánchez Saavedra, María Del PilarGabriela Vollet, MarsonSanchéz-Burgos, Jorge AlbertoGallardo-Rodríguez, Juan JoséSánchez-Fernández, Elena M.Gallo, AlessandraSandjo, Louis PergaudGallo, MonicaSandri, GiuseppinaGalvez Llompart, MariaSanjeewa, Kalu Kapuge AsankaGaly-Fauroux, IsabelleSanniyasi, ElumalaiGama, SofiaSansone, AnnaGan, Mao-luoSantos, Mario Ferreira ConceiçãoGanesan, A.Saravana, Periaswamy SivagnanamGanesan, Dr. Raja NSardari, Roya R. R.García García, PatriciaSardo, AngelaGarcía, CarlosSarragiotto, MariaGarcía-Arrarás, José E.Satake, MasayukiGarcía-García, PilarSato, KenjiGarcia-Jimenez, PilarSato, YuichiroGard, PaulSauvage, ThomasGardikis, KonstantinosSavion, NaphtaliGasparri, FabioSayed, AhmedGassaway, Brandon MarkSayed, Ahmed M.Gautam, VibhavSchaal, WesleyGenovese, GiuseppaSchäberle, TillGerbaux, PascalSchikov, AlexanderGerdol, MarcoSchillaci, DomenicoGhimire, Bimal KumarSchling, PetraGiannetto, DanielaSchröder, Hans-christophGill, TomSchröder, Heinz C.Giordano, DanielaSchroeder, ChristinaGirich, ElenaSciani, Juliana MozerGlossman-Mitnik, DanielScipione, LuigiGlukhov, EvgeniaSébastien Wieckowski, SébastienGoda, Ahmed E.Secenji, AleksandarGodyń, JustynaSedlak, MilosGolinelli, Marie-PierreSeedevi, PalaniappanGomaa, MohamedSelvaraj, GurudeebanGomez Estaca, JoaquinSemenycheva, Ludmila L.Gómez Pinchetti, Juan LuisSeo, Jung-KilGómez-Zorita, SaioaSetiawan, AndiGoncalves, OlivierSeto, ShigekiGonzalez, Miryam CriadoSetzer, William N.González-Durruthy, MichaelShaaban, MohamedGorbushin, AlexanderShaala, Lamiaa A.Górska, SabinaShah, Muhammad AjmalGouda, MostafaShah, Muhammad DawoodGovindan, Dr. NatanamurgarajShaish, AvivGoyette-Desjardins, GuillaumeSharma, KavitaGrauso, LauraShastry, Rajesh P.Green, BenShen, Chun-YanGrkovic, TanjaShi, DayongGroß, HaraldShi, YunGrosso, ClaraShih, Wen-LingGu, WenjiaShikov, Alexander N.Guan, Li-PingShin, Hee JaeGuil-Guerrero, José LuisShinomura, TomokoGulzar, SalmanShintyapina, Alexandra B.Gurgu, Leontina C.Šic Žlabur, JanaGushina, IrinaSiddique, HifzurGustafson, KirkSieber, SimonGuzik, MaciejSikazwe, DonaldGuzmán, EduardoSikorskaya, TatyanaGuzman, FannySilchenko, Alexandra S.Hahn, AndreasSilipo, AlbaHan, JingSilva, MarisaHan, Jong WonSilva, Simone S.Han, WenjunSilva, TiagoHan, YanhuiSilva-Abreu, MarcelleHandayani, DianSilvestre, WendelHanif, NovriyandiŠimat, VidaHanisch, Franz-GeorgSimpson, Bradley S.Hanna, HarantSimpson, Thomas J.Hannan, Md AbdulSims, Ian M.Hansen, EspenSineiro, JorgeHarayama, ShigeakiSingh, Sitanshu S.Harder, JensSkov, JakobHarrison, Paul H. M.Sliwka, Hans-RichardHarvey, JoanneSmith, JulietteHasan, ImtiajSmith, KerryHasegawa, YasushiSmułek, WojciechHashimoto, MasaruSnowden, TimothyHassan, Mohamed A.Söderhäll, KennethHassan, Syed Shams UlSoengas, Raquel G.Hassan, Walid M. I.Solano, FranciscoHaug, TorSolcan, CarmenHavlik, IvoSoldatou, SylviaHe, FeiSolovchenko, AlexeiHegazy, Mohamed-Elamir F.Song, FuhangHelbert, WilliamSong, ShuangHenríquez, VitaliaSong, XiaojinHeo, Seong-YeongSong, YongxiangHeo, Soo-JinSoowannayan, ChumpornHernández Daranas, AntonioSorg, OlivierHernandez-Prieto, Miguel A.Soriano-Romaní, LauraHerrin, DavidSoriano-Ursúa, Marvin A.Hess, PhilippSorrentino, AngelaHeymann, DominiqueSosa, SilvioHigashi, NobuakiSotelo-Mundo, Rogerio R.Hinkley, SimonSoudant, PhilippeHitora, YukiSouto, JaneusaHo, MinghuaSoveral, GraçaHolland, Christopher WilliamSprynskyy, MyroslavHolmes, MichaelSreenivasmurthy, Sravan GopalkrishnashettyHonda, MasakiStefan, SimmHoseinifar, Seyed HosseinStenstrøm, YngveHosomi, RyotaStoskopf, MichaelHotos, George N.Sukweenadhi, JohanHristova, Rosalina StanchevaSun, FengjieHsieh, YvesSun, ZhaoxiHsu, Chih-pingSundaram, BhagavathiHu, Jin-JiaSung, Ping-JyunHu, ZhengxiSuomela, Jukka-PekkaHuang, Shih-YiSurup, FrankHuang, Wen-ChungSuttajit, MaitreeHuang, XueshiSyama Dayal, JagabattullaHuber, MichaelSzabó, ÉvaHudmon, AndySzakiel, AnnaHui, Chang-YeSzilágyi, AndrásHurst, Brett L.Tabanca, NurhayatHurtado Oliva, Miguel ÁngelTacer Caba, ZeynepHussain, Ali A.Tafvizi, FarzanehHussain, HidayatTailhades, JulienHutamekalin, PilaiwanwadeeTaira, JunseiI. Kalinin, VladimirTakagi, YasuakiIacob, Andreea-TeodoraTakahashi, KoretaroIbrahim, SabrinTakaichi, ShinichiIchikawa, NatsukoTakano, KatsuraIelciu, IrinaTakegahara, NorikoIijima, NoriakiTakeno, SachioIjaz, ShakeelTakeshita, SatoshiIlag, Leodevico L.Talamonti, Emanuelaİlbasmiş Tamer, SibelTalens-Perales, DavidImperatore, ConcettaTammam, Mohamed A.Imperatore, RobertaTampa, MirceaIndurthi, DineshTanaka, JunichiIngham, Colin J.Tanase, CorneliuIqbal, DanishTang, Bor LuenIsaeva, Marina P.Tang, Chuan-HoIshaq, Muhammad SaqibTangadanchu, Vijai Kumar ReddyIslam, Md. AmirulTangerina, Marcelo Marucci PereiraIsmael, M. IsabelTankiewicz-Kwedlo, AnnaItabaiana, IvaldoTanzi, Maria CristinaItoh, TakafumiTarman, KustiariyahItoi, ShiroTartarotti, BarbaraIuco Castro Da Silva, IgorTaşdemir, DenizIvanchina, NataliaTaskın-Tok, T.Jabir, Majid S.Taylor, MatthewJachak, GorakhnathTéletchéa, StéphaneJanowski, MiroslawTengku Muhammad, Tengku SifzizulJaradat, NidalTerrasson, VincentJaramillo Flores, María EugeniaTersariol, Ivarne L. S.Jarończyk, MałgorzataTeruya, KiichiroJaudzems, KristapsTeta, RobertaJayaraman, GurunathanTHAKUR, NarsinhJayawardena, Thilina U.Thibault, DelphineJe, Jae-YoungThomas, JohnJégou, CamilleThomas, OlivierJembrek, Maja JazvinšćakThomaz, Douglas VieiraJensen, PaulTidgewell, KevinJesionowski, TeofilTietjen, IanJian, HuahuaTilvi, Supriya ShetJiang, BoTimoshenko, Alexander V.Jiang, GuangdeTischler, DirkJiao, GuanglingTius, MarcJiménez, CarlosToida, ToshihikoJimenéz, Elsie C.Tomaszewska, EwaJimenez, Paula ChristineTomić, SimonidaJiménez-Tenorio, ManuelTong, HaibinJin, ZhouTosti, ElisabettaJoão, CotasTouloupakis, EleftheriosJoão, SilvaTousif, Muhammad ImranJoo, Hong-GuTran, TrongJorge, ErikaTrianto, AgusJouanneau, DianeTrif, MonicaJung, Jee-hyungTripathi, AshootoshJung, Won-KyoTsai, Yow-FuJunker, JochenTsakovska, IvankaKaas, Quentin K.Tsatsanis, ChristosKabeya, NaokiTsetlin, VictorKacem, AdnenTsetlin, Victor I.Kadokawa, Jun-ichiTudela, JoseKadota, IsaoTurner, AndrewKageyama, HakutoTuvikene, RandoKahramanoğulları, OzanTziveleka, Leto-AikateriniKalinin, VladimirUeno, AkioKalinin, Vladimir I.Ulasov, IlyaKamal, SitiUlber, RolandKang, Jeong-HunUllah, RiazKaraman, MajaUmezawa, KazuoKarpinski, Tomasz M.Umezawa, TaikiKashman, YoelUpadhyay, SantoshKasrati, AyoubUroš, AndjelkovićKašuba, VilenaUsami, YoshihideKaszowska, MartaUsov, Anatolii IKatapodis, PetrosUthappa, Uluvangada ThammaiahKatikou, PanagiotaUzunov, BlagoyKatranidis, AlexandrosVaiano, FabioKawamura, AkiraValado, AnaKawano, Daniel FábioValdés, AlbertoKawano, ShigeyukiVale, CarmenKazachenko, AlexandrVale, PauloKeglevich, GyörgyValenzuela Soto, Elisa MiriamKeiffer, Timothy R.Van Den Berg, BernardKelly, MichelleVanic, ZeljkaKem, WilliamVarvaresou, AthanasiaKempken, FrankVázquez-Flota, FelipeKerch, GarryVélaz, ItziarKerdjoudj, HalimaVenmathi Maran, Balu AlagarKermanshahi-pour, AzadehVerdes, AidaKeyzers, RobVergara, DanieleKeyzers, RobertViault, GuillaumeKhalil, ZeinabVieira, HelenaKhalloufi, Fatima ElVijayaraghavan, PonnuswamyKhan, FazlurrahmanVil’, Vera A.Khan, HaroonVilariño, NataliaKhan, ShahVilla, CarlaKharat, Arun S.Vinayagam, RamachandranKhlebnikov, Andrei I.Vinogradov, EvgueniiKhoo, CheangVistoli, GiulioKhotimchenko, Maxim Y.Vlachova, ViktorieKikionis, StefanosVolkova, Tatyana V.Kikukawa, HiroshiVolod’ko, Aleksandra V.Kim, Chong-TaiVon Kriegsheim, AlexKim, ChoonVrana Špoljarić, IvnaKim, Chu-YoungWakimoto, ToshiyukiKim, DonghwaWalker, AndrewKim, EuikyungWang, Bin-GuiKim, Hei-sungWang, BoKim, HiyoungWang, ChunguangKim, Hyoung GyuWang, HaiboKim, Hyun-oukWang, KuiwuKim, In JungWang, WeizhongKim, Jae-IlWang, YanboKim, Ok-KyungWang, YanchaoKimbaris, AthanasiosWang, Yu-MingKinnel, RobinWang, ZiwenKiper, Aytuğ K.Wanyonyi, StephenKishimura, HidekiWatabe, ShugoKispert, Lowell D.Watts, Jeffrey L.Kita, MasakiWefers, DanielKiuru, PaulaWeger, HaroldKlar, AgnesWeinberger, FlorianKlegerman, Melvin E.Welker, MarkKleinpeter, ErichWhite, LindseyKloareg, BernardWichmann, JulianKnölker, Hans-JoachimWilliams, PhilipKobayashi, DaisukeWójciak, MagdalenaKobayashi, JunWójciak-Kosior, MagdalenaKobori, ToshiroWojciezsyńska, DanutaKoh, Cho YeowWong, Kah HuiKoike, KenzoWong, Tin WuiKoksharova, Olga A.Wood, Paul L.Kolesnikova, Sophia A.Wróbel-Biedrawa, DagmaraKolobov, Alexandr AlexandrovichWu, JingKolotova, DariaWu, MingyiKomaki, HisayukiWu, QihaoKommineni, NagavendraWu, Shih-HsiungKonturek, Peter ChristopherWu, Tzong-YuanKoo, Song YiWu, Tzu-HuaKorolkova, Y. V.Wu, ZhenbingKorshun, Vladimir A.Xiao, MeitianKos, BlaženkaXiao, TianKose, AyseXu, DongboKoshino, HiroyukiXu, JiakeKosma, PaulXu, NianjunKotake-Nara, EiichiXu, XueqingKotoku, NaoyukiXu, Zhi PingKoua, DominiqueYabuta, YukinoriKowalska, JustynaYadav, VikasKozanecki, MarcinYadava, Ramesh S.Kozlov, SergeyYalçın, SibelKozłowska, JustynaYamamoto, KazuhiroKról, EwelinaYamano, YoshiKrug, DanielYang, Jin-YoungKryshchyshyn, Anna P.Yang, QiaoKudryavtsev, Denis S.Yang, Se-RanKulikovskiy, Maxim S.Yaoita, YasunoriKumar, RajYeap, Swee-KeongKuranova, I. P.Yeh, Tai-ShengKuroda, ChiakiYeom, JunseokKusaykin, Mikhail I.Yokose, SatoshiKutryb-Zaja̧c, BarbaraYoo, KeunjeKwak, Ho SeokYoshida, MasahitoKyeremeh, KwakuYotsu-Yamashita, MariLa Clair, JamesYoussef, DiaaLa Regina, GiuseppeYoussef, FadiaLabes, AntjeYu, HuahuaLacret, RodneyYu, JianLade, Harshad S.Yudin, VladimirLaezza, AntonioYurchenko, EkaterinaLai, Kuei-HungZaccone, GiacomoLamari, Fotini N.Zaitone, SawsanLauder, BobZaitsev, Kirill V.Lavison-Bompard, GwenaelleZammuto, VincenzoLavoie, SergeZamora-Bustillos, RobertoLaw, Jia XianZarrouk, AmiraLazarovici, PhilipZavistanaviciute, PaulinaLazzari, ElisaŻbikowska, AnnaLe Bideau, FranckZeimaran, EhsanLeao, JoséZelepuga, ElenaLe-Deygen, Irina M.Zeng, HaisuLee Chang, Kim JyeZhang, ChangshengLee, Bong HoZhang, DongluLee, ChowZhang, FumingLee, HyeyoungZhang, GuojianLee, Hyi-SeungZhang, HaiboLee, I-TaZhang, HuaLee, JihoonZhang, JunzengLee, MinaZhang, KuiLee, Pyeong ChunZhang, LanLee, VictorZhang, LiLee, Yeon-JuZhang, QuanbinLegros, ChristianZhang, WenjunLehocký, MariánZhang, YonghongLengerer, BirgitZhang, Yong-HuiLeón, RosaZhang, Zhi-ZhenLeonardi, SalvatoreZhao, XueLeone, SerenaZhdanov, Dmitry D.Leong, Yoong KitZhorov, Boris S.Leow, ChiuanZhou, XiaoqiuLeshchenko, ElenaZhou, XuefengŁęska, BogusławaZhou, Ying-GuoLetsiou, SophiaZhu, KuiLewinska, AnnaZhu, LifengLewis, RichardZhu, YiguangLeychenko, ElenaZhuang, YongliangLeyton, AllisonZhuravleva, Olesya I.Li, FuliZidovec Lepej, SnjezanaLi, HanxiangŻołnowska, BeataLi, HaoZouridakis, MariosLi, Hou-JinZubía, EvaLi, JianZuev, Yu. F.Li, Jie



